# Loss of *rps9* in Zebrafish Leads to *p53*-Dependent Anemia

**DOI:** 10.1534/g3.119.400585

**Published:** 2019-10-16

**Authors:** Cheng Chen, Haigen Huang, Ruibin Yan, Shuo Lin, Wei Qin

**Affiliations:** * State Key Laboratory of Chemical Oncogenomics, Key Laboratory of Chemical Genomics, Peking University Shenzhen Graduate School, Shenzhen, 518055, China and; † Department of Molecular, Cell and Developmental Biology, University of California, Los Angeles, 90095 CA

**Keywords:** ribosomal protein, anemia, *rps9*, *p53*, zebrafish

## Abstract

Ribosome is a vital molecular machine for protein translation in the cell. Defects in several ribosomal proteins including RPS19, RPL11 and RPS14 have been observed in two types of anemia: Diamond Blackfan Anemia and 5q- syndrome. In zebrafish, deficiency of these ribosomal proteins shows similar anemic phenotype. It remains to be determined if any other ribosome proteins are similarly involved in regulating erythropoiesis. Here we generated mutations in zebrafish *rps9*, a rarely studied ribosomal protein gene, and investigated its function. Analysis of this mutant demonstrates that *rps9* disruption leads to impairment of erythrocyte maturation, resulting in anemia. In addition, the overall phenotype including the anemic state is *p53*-dependent in *rps9* mutants. Furthermore, this anemic state can be partially relieved by the treatment of L-leucine, and dexamethasone, which have been previously used in rescuing the phenotype of other ribosomal protein mutants. Finally, by comparing the phenotype, we show that there are considerable differences in morphology, cytomorphology, and hemoglobin levels for four ribosomal protein mutants in zebrafish. Based on the observed difference, we suggest that the level of anemic severity correlates with the delayed status of erythrocyte maturation in zebrafish models.

As a vital machine for mRNA translation, ribosome comprises a large 60S subunit containing 25S, 5.8S and 5S rRNA with 46 ribosomal proteins and a small 40S subunit containing 18S rRNA with 33 ribosomal proteins (Thomson *et al.* 2013). Ribosomal proteins play a general but essential role in protein translation and ribosome assembly. However, ribosomal proteins also have specific function such as interaction with *p53* to transfer ribosomal stress to the cell, which elicits cell cycle arrest and apoptosis (Zhou *et al.* 2015).

Due to the pivotal roles of the ribosome, defects in ribosomal proteins can cause many diseases. Several defective ribosomal proteins have been discovered as causal factors of the human disease Diamond-Blackfan anemia, which is a type of anemia characterized by macrocytic anemia and bone defects. Notably, only one deficient ribosomal protein, RPS14, is identified in 5q- syndrome, which is another type of anemia diagnosed with macrocytic anemia and micromegakaryocytes accompanied with a low risk of developing into acute myeloid leukemia (Narla and Ebert 2010). Early studies in zebrafish have demonstrated that *p53* activation induced by ribosomal protein deficiency plays a major role in causing anemia, although other *p53*-independent signaling pathways are also involved in these disorders (Danilova *et al.* 2008; Torihara *et al.* 2011; Provost *et al.* 2012).

Previous genetic studies of ribosomal proteins in model animals such as zebrafish have focused on a few ribosomal protein genes including *rps19*, *rpl11*, and *rps14* due to their prior identification in human diseases (Danilova *et al.* 2011; Jia *et al.* 2013; Zhang *et al.* 2013; Zhang *et al.* 2014; Ear *et al.* 2016). It remains to be determined if the function of other ribosomal protein genes is also involved in regulating erythropoiesis. This question was addressed by transient gene knockdown using morpholino (MO) targeting nearly two dozen of ribosomal protein genes (Uechi *et al.* 2006; Uechi *et al.* 2008). Uechi *et al.* discovered that various degrees of defects in morphology and erythropoiesis were induced by knockdown of different ribosomal protein genes. These results suggest that not all ribosomal proteins are equally involved in regulating erythropoiesis. However, it has been recently appreciated that phenotypic results obtained from studies of morphants and genetic mutants often have discrepancies (Kok *et al.* 2015). Since *p53* activation, which plays a role in phenotype of ribosomal deficiency, can be non-specifically induced by morpholino (Robu *et al.* 2007), it is better to interrogate ribosomal function using genetic mutants for consistency.

*rps9* is a ribosomal protein contained in the 40S subunit of the ribosome. Sporadic studies on *rps9* have demonstrated its interaction with nucleophosmin, its role in normal cell proliferation as well as regulation in osteosarcoma cell growth (Lindström and Zhang 2008; Lindström 2012; Cheng *et al.* 2017). However, no research on the organism level has been reported to elucidate the function of *rps9 in vivo*. In this study, we generated *rps9* mutants by CRISPR/Cas9 gene targeting in zebrafish. Detailed analyses showed that mutation in *rps9* could lead to anemia, accompanied by severe morphological abnormalities. This anemic phenotype was due to a block of the terminal maturation of erythroid cells. Further study revealed that the anemia was largely dependent on *p53* signaling pathway. Treatment of previously used agents including L-leucine and dexamethasone was able to alleviate the anemia phenotype in *rps9* mutant. Moreover, the comparison of different ribosomal protein mutants revealed considerable differences in morphology, erythroid cytomorphology, and blood level for different mutants. Overall, our study suggests that *rps9* can be a candidate for human anemia disease.

## Materials and Methods

### Zebrafish strains and lines

All zebrafish experiments were approved by Institutional Animal Care and Use Committee of Peking University and UCLA. *rps9* mutants were generated using CRISPR-Cas9 method in wild-type (AB) strain, while *rpl11*, *rps14*, and *rps19* mutants were previously described (Danilova *et al.* 2011; Zhang *et al.* 2014; Ear *et al.* 2016).

### mRNA and gRNA synthesis, genotyping

pT3TS-nCas9n plasmid (addgene, plasmid #46757) for zebrafish was linearized with *Xba*I, mRNA was obtained through *in vitro* transcription using linearized plasmids with T3 mMESSAGE Kit (Ambion). For *rps9* mRNA synthesis, coding sequence was amplified using cDNA and cloned into pCSDest2 using gateway technology (Thermo Fisher), then mRNA was transcribed after plasmid linearization using SP6 mMESSAGE Kit (Ambion). Rescue experiment was performed by injecting 400 pg *rps9* mRNA to the embryos at one cell stage. For gRNA synthesis, DNA template was amplified using gene specific oligo (rps9 guide F: TAATACGACTCACTATAGCGTATTGGAGTGCTGGATGGTTTTAGAGCTAGAAATAGC), then gRNA was transcribed *in vitro* using MAXIscript T7 kit (Ambion) and purified using MicroRNA Isolation Kit (BioChain USA). 200 pg of Cas9 mRNA and 100 pg of gRNA were mixed and injected into embryos at the one-cell stage. For genotyping, PCR products were amplified from genomic DNA and subjected to electrophoresis using 8% TBE-PAGE gel with the following primers: *rps9* Fwd: 5′-TTCCTCGTCAATGAGGCCAT-3′ and *rps9* Rev: 5′-CTTTGCACATGTAGTTAGCA-3′. PCR products showing positive bands were further cloned and sequenced.

### Wright-Giemsa staining

Embryos were anesthetized with tricaine, then immersed in PBS/40% FBS (Hyclone) mixed with a final concentration of 5 mM EDTA (Corning). Blood cells were collected by stabbing the heart using a micro-injection needle. Collected cells were transferred on the slides and air dry. After fixing in the methanol for 5 s, cells were soaked in Wright-Giemsa staining solution for 15 s. Finally, slides were rinsed with deionized water. Images were taken with AxioPlan2 after air dry.

### In situ hybridization, TUNNEL staining, o-dianisidine staining

cDNA fragment for *rps9* was cloned into pUC19 plasmid (Takara) using *rps9*-probeF: 5′-GGTACCTGTTCGAGCCTGACACGGAC-3′ and *rps9*-probeR:5′-TAATACGACTCACTATAGCAAGCACTGCTGAGTCCCT-3′. Probes for other genes were used as previously described (Danilova *et al.* 2011; Ear *et al.* 2016). Whole mount *in situ* hybridization was performed as described (Thisse and Thisse 2008).

Embryos for TUNNEL staining were stored as described in whole mount *in situ* hybridization. Subsequent TUNNEL staining was performed on the embryos following the manufacturer’s protocol (Roche, In Situ Cell Death Detection Kit, TMR red).

For o-dianisidine staining, embryos were collected and fixed at room temperature for 2 h using 4% paraformaldehyde (PFA) in PBS buffer after dechorionation. After the removal of PFA by PBS wash, embryos is incubated in the o-dianisidine (Sigma) staining solution in the dark for 30 min followed by PBS wash. Embryos were then bleached for 20 min. Finally, embryos were immediately imaged after washed by PBS.

### Real-time quantitative PCR

Total RNA was isolated using TRIzol Reagent (Life Technologies) from pool of 30 embryos according to the manufacturer’s directions. cDNA was made using ProtoScript II Reverse Transcriptase and random hexamer primers (New England Biolabs). Quantitative PCR was performed on Stratagene Mx3005P (Agilent Technologies) using Luna Universal qPCR Master Mix (New England Biolabs). Each experiment was performed in triplicate. Primers for *p53*, *mdm2*, and *p21* are previously described (Ear *et al.* 2015), and primers for *rps9* are as follows: *rps9*-oligoF: 5′-AGAAGGATCCTAAGCGTCTC-3′, *rps9*-oligoR: 5′-CTCTCCAAGAAATCCTCCAC-3′.

### Data availability

All data generated or analyzed during this study are included in this article and its supplementary information files. Figure S1-S2 contains protein alignment and gene targeting illustration. Figure S3-S7 and Movie S1-2 contain blood related information. Figure S8 contains vessel related information. File S1 contains a full description of these 8 supplementary figures. Supplemental material available at figshare: https://doi.org/10.25387/g3.9948260.

## Results

### Deficiency in rps9 leads to morphological defects

Located in chromosome 16, *rps9* gene is highly conserved between human and zebrafish and their proteins share 95% of amino acid similarity (Figure S1). By targeting *rps9* with CRISPR/Cas9, three mutant lines were generated. All these three mutant lines had a small deletion in the third exon of *rps9* locus. Specifically, two frame-shift mutant lines, *rps9^la491^* and *rps9^la490^*, have alleles predicted to encode truncated polypeptides and one mutant line, *rps9^la489^*, has an allele producing a protein with one amino acid deletion and a leucine changed to histidine (Figure S2, A and B). Three mutant lines were outcrossed to wildtype fish and maintained for further experiments.

**Figure 1:**
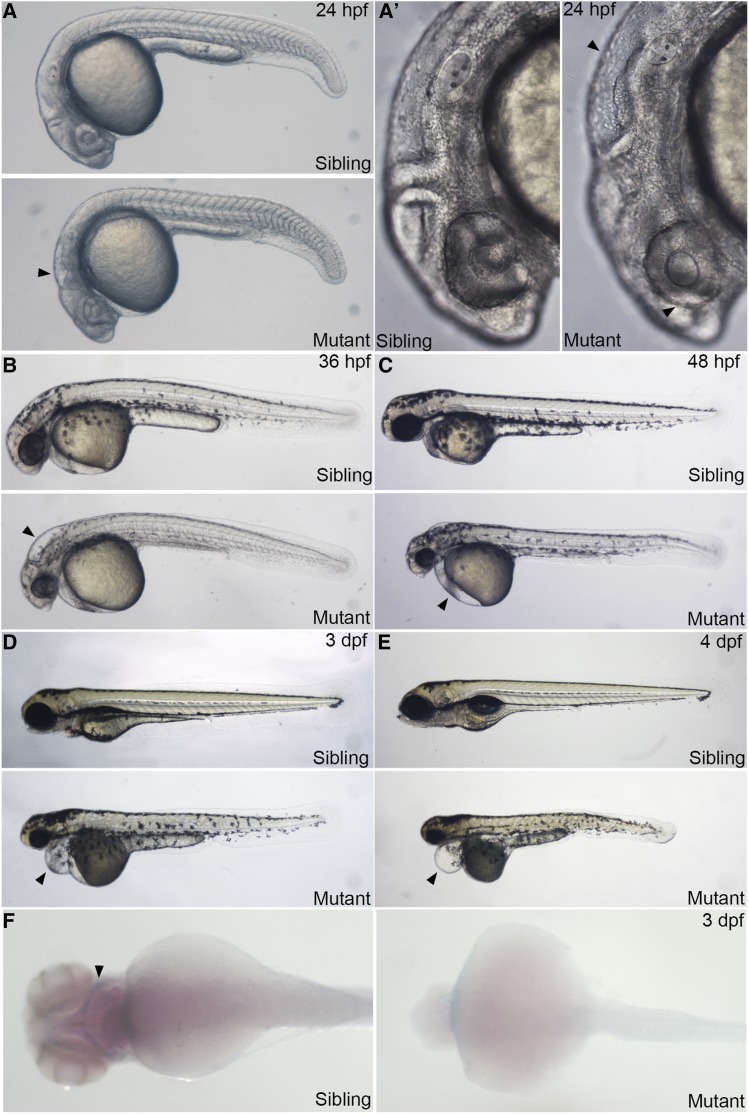
Embryos with *rps9* deficiency display morphological abnormalities. (A-E) Overall morphological phenotype of *rps9* deficient embryos. At 24 hpf (A and A’), smaller eyes and abnormal hindbrain (arrowhead) appear in the head region. By 36 hpf (B), enlarged hindbrain (arrowhead) becomes apparent and pigment is reduced. From 48 hpf to 4 dpf (C-E), edema (arrowhead) is gradually developed in the pericardial region, accompanied with shortened trunk. (F) Cartilage fails to grow in the pharyngeal arch in *rps9* mutant embryos.

No notable abnormality was observed in offspring from in-crossing the *rps9^la489^*/*rps9* mutant line. When *rps9^la491^*/*rps9* and *rps9^la490^*/*rps9* adult fishes were in-crossed, embryos presumably with homozygous mutation started to show notable abnormality from 21 hr post fertilization (hpf). By 24 hpf, aplasia was seen in the head in *rps9*-deficient embryos, indicated by small eyes and enlarged hindbrain ventricles (Figure 1A and 1A’). Smaller eyes and enlarged hindbrain were apparent at 36 hpf, accompanied by reduction of pigment (Figure 1B). From 48 hpf to 4 days post fertilization (dpf), the pericardium gradually became enlarged and finally developed edema. The body length also decreased and the trunk displayed apoptosis (Figure 1, C-E). The homozygous embryos eventually died at 5 dpf. Furthermore, the formation of cartilage was blocked when examining with alcian blue staining at 3 dpf, indicated that cartilage development was disrupted in *rps9* mutants (Figure 1F). To verify that the morphologically abnormal embryos were linked to *rps9* mutation, we genotyped them by direct sequencing. Sequencing results showed that the embryos (N > 5) with morphological defects all had the identified indels in the *rps9* locus (Figure S2C). This study confirmed a null deletion in the *rps9* gene is associated with the observed phenotype of *rps9^la491^* and *rps9^la490^*.

**Figure 2:**
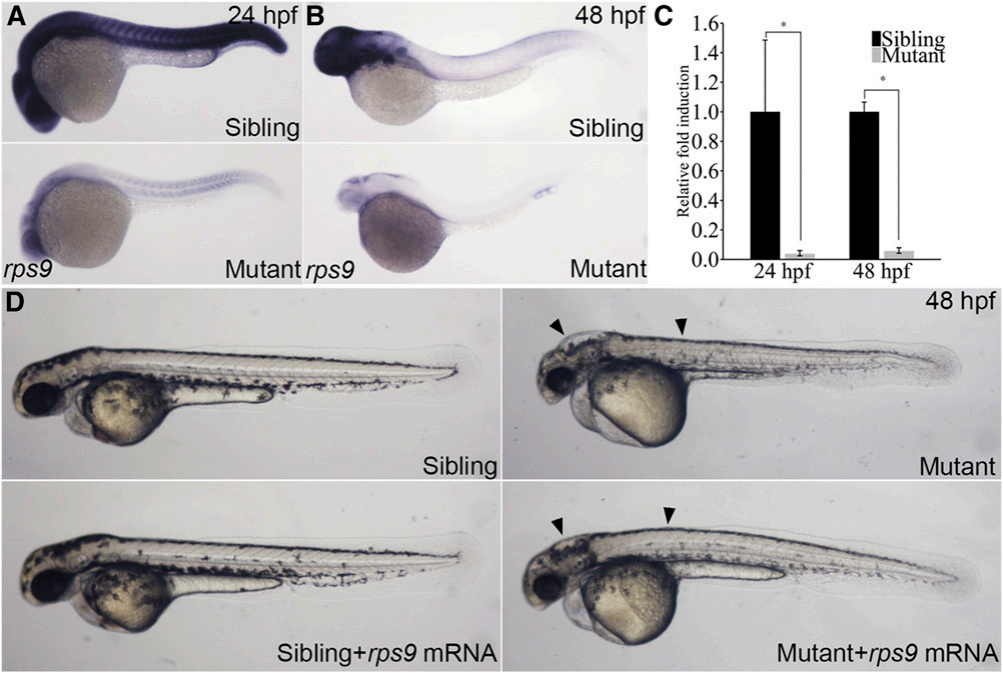
Phenotype of *rps9* deficient embryos is partially rescued by *rps9* mRNA injections. (A-B) Whole mount *in situ* hybridization for *rps9* in mutants and siblings at 24 hpf or 48 hpf. (C) qPCR analysis of *rps9* transcript levels. *, *P* < 0.05. (C) *In vitro* transcribed *rps9* mRNA (400 pg) partially rescues phenotype in *rps9* mutants. Arrowhead indicates morphological improvement in the hindbrain and trunk.

To determine expression level and patterns of *rps9*, we examined its expression by whole-mount RNA *in situ* hybridization. At 24hpf, the wild type sibling showed strong expression throughout the body while the mutant displayed a dramatic reduction in expression levels (Figure 2A). Similarly, a remarkable decrease of gene expression was observed in the *rps9* deficient embryos by 48 hpf (Figure 2B). This reduction of *rps9* transcript in the mutant was also revealed quantitatively by real-time PCR (Figure 2C). To further confirm that reduction of *rps9* transcript level was responsible for the morphological abnormalities in mutants, we conducted a rescue experiment using *in vitro* transcribed *rps9* mRNA. Indeed, injection of *rps9* mRNA to the mutant embryos improved morphology both in the hindbrain and trunk (Figure 2D). Altogether, these results established that the morphological defects seen in the mutant were due to the deficiency of *rps9*.

### rps9 is required for normal maturation of erythroid cells

**Figure 3:**
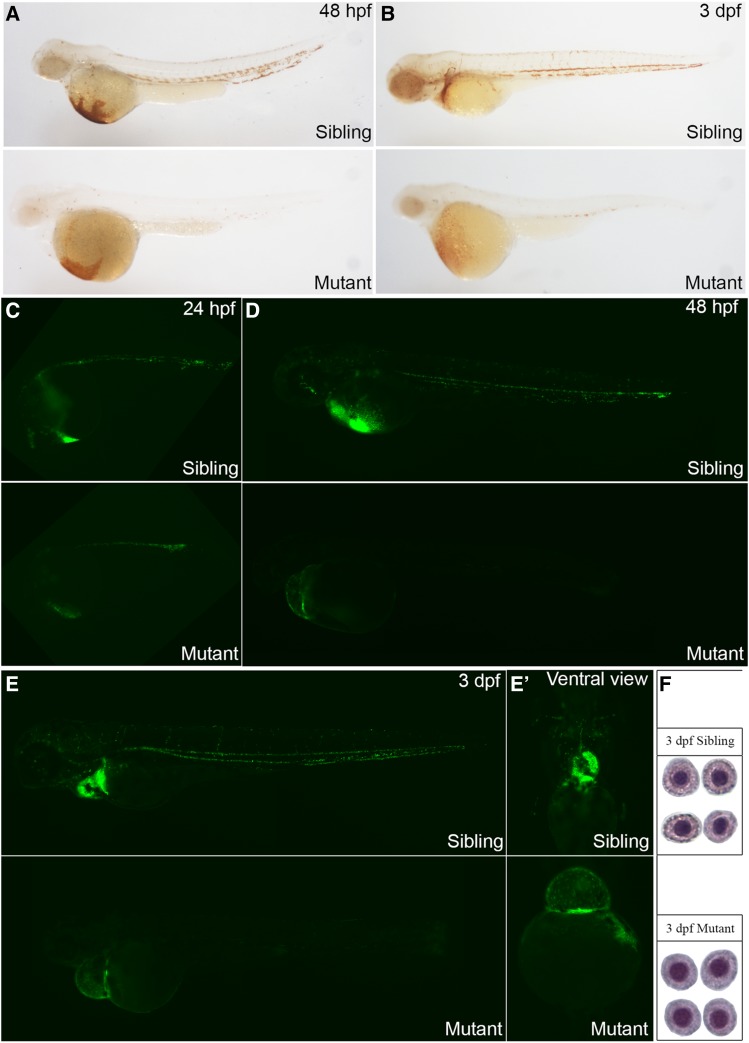
Deficiency in *rps9* induces anemia in mutant embryos. (A-B) Detection of hemoglobin levels by o-dianisidine staining for *rps9* in both siblings and mutants at 48 hpf and 3 dpf. (C-E, E’) Detection of erythroid cells by crossing *rps9* mutant into *LCR:EGFP* transgenic fish line. At 24 hpf, the number of blood cells are reduced slightly; At 48 hpf and 3 dpf, erythrocytes are almost deprived in the trunk, and only a small amount of red blood cells remain in the cardiac region. (F) Wright-Giemsa staining of erythroid cells at 3 dpf in sibling and mutant.

We next evaluated whether the development of red blood cells was affected by *rps9* deficiency in the zebrafish mutants. We found that, at both 48 hpf and 3 dpf, o-dianisidine staining level in embryos was severely reduced in the mutants (Figure 3, A and B) and this reduction in hemoglobin levels could be partially restored by the injection of *rps9* mRNA (Figure S3), implying that deficiency in *rps9* was responsible for the anemic phenotype. To evaluate numbers of red blood cells, we mated *rps9* mutant line into the *LCR2:EGFP* transgenic fish line, whose red blood cells were tagged with EGFP. A moderate decrease of the EGFP positive cells was revealed between the sibling and mutant embryos at 24hpf, and the decline became significant at 48 hpf (Figure 3, C and D). At 3 dpf, EGFP positive cells were only found at the cardiac region (Figure 3, E and E’). Furthermore, blood circulation was diminished at 24 hpf and gradually lost due to the lack of blood cells and malfunctioned heart (Movie S1 and S2).

To detect the cytomorphological change of the erythroid cells in *rps9* deficiency, we did a Wright-Giemsa staining at 3 dpf. Based on the size and shape of the nucleus as well as staining of the plasma, the developmental stage of the erythroid cells can be identified (Qian *et al.* 2007). In *rps9* mutant, the erythroid cells remained at the polychromatophilic stage, which was usually seen at 2 dpf (Figure 3F). This developmental delay of erythrocyte suggests that the maturation of erythrocyte is impaired in *rps9* deficient embryos.

**Figure 4:**
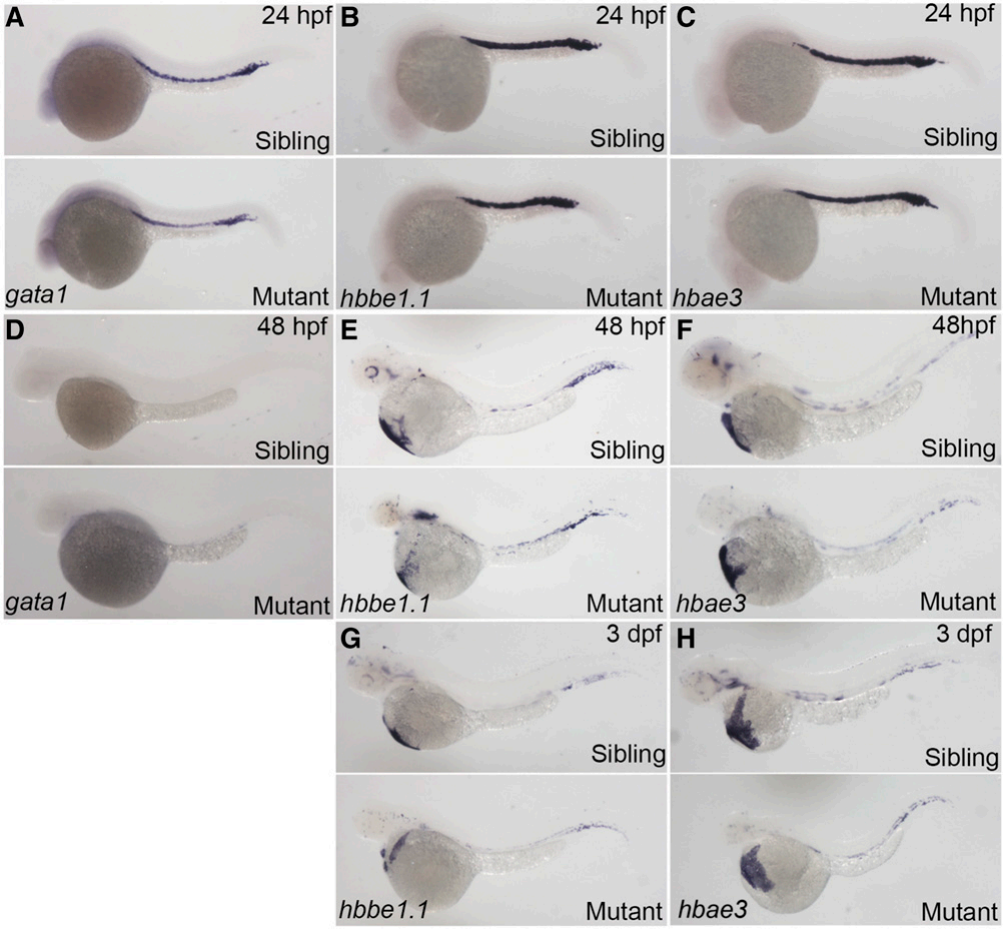
No obvious change was seen for expression of erythroid specific markers in *rps9* mutants. (A-F) Expression levels of *gata1*, *hbbe1.1*, and *hbae3* in mutants remain unchanged at 24 hpf (A-C) and 48 hpf (D-F). (G-H) Because of the shortened size of the mutant embryos, slight decrease in *hbbe1.1* and *hbae3* is seen at 3 dpf.

We then accessed the effect of *rps9* disruption on erythropoiesis in mutant embryos by detecting the gene expression pattern of three erythroid markers at several developmental stages. As one of the markers for the specification of erythroid lineage, *gata1* was used to detect the early stage of erythropoiesis. Two other globin transcripts, *i.e.*, *hbbe1.1*, and *hbae*, can be used to mark the mature red blood cells. At 24 hpf, the expression of *gata1*, *hbbe1.1*, and *hbae3* in mutant embryos was indistinguishable from sibling control embryos, suggesting the specification step of erythroid cells is not affected (Figure 4, A-C). At 48 hpf, both mutant and sibling control embryos displayed a faint expression of *gata1*, and the expression of *hbbe1.1* and *hbae3* remained mostly unchanged (Figure 4, D and F). Additionally, the expression of *hbee1.1* and *hbae3* appeared slightly decreased at 72 hpf due to the shrunken size of mutant embryos (Figure 4, G and H). These results suggest that overall erythroid gene transcription is not significantly downregulated.

To investigate the effect of *rps9* deficiency on overall hematopoiesis, we analyzed other non-erythroid markers. A similar expression pattern was observed for three markers of myeloid cells, namely, *l-plastin*, *mpx*, and *pu.1*, indicating that the specification of primitive myeloid cells is not affected (Figure S4, A-C). Likewise, the development of hematopoietic stem cell is not affected as there was no considerable change for the expression level for *scl* and *c-myb*, markers for the hematopoietic stem cell emergence (Figure S4, D and E). However, expression of *rag1* decreased significantly, suggesting that lymphoid development is disturbed (Figure S4F).

Finally, the expression pattern for two other ribosomal protein genes was also detected to check the expression change for other ribosomal protein genes. The expression of *rpl11* and *rps14* shares a similar pattern between the mutant and wildtype sibling control, suggesting that the regulation for other ribosomal protein genes is unaffected upon the deficiency of *rps9* (Figure S5).

### rps9 deficiency leads to p53 upregulation and downregulation of p53 can largely rescue the phenotype

The morphological defect is usually associated with an abnormal increase in the levels of apoptosis. To determine the level change of apoptosis, we conducted a TUNNEL staining for both the mutant and wildtype sibling control. An elevation of the apoptosis level was indeed detected at all time points analyzed, confirming that morphological defect is likely caused by increased activity of apoptosis (Figure S6).

**Figure 5:**
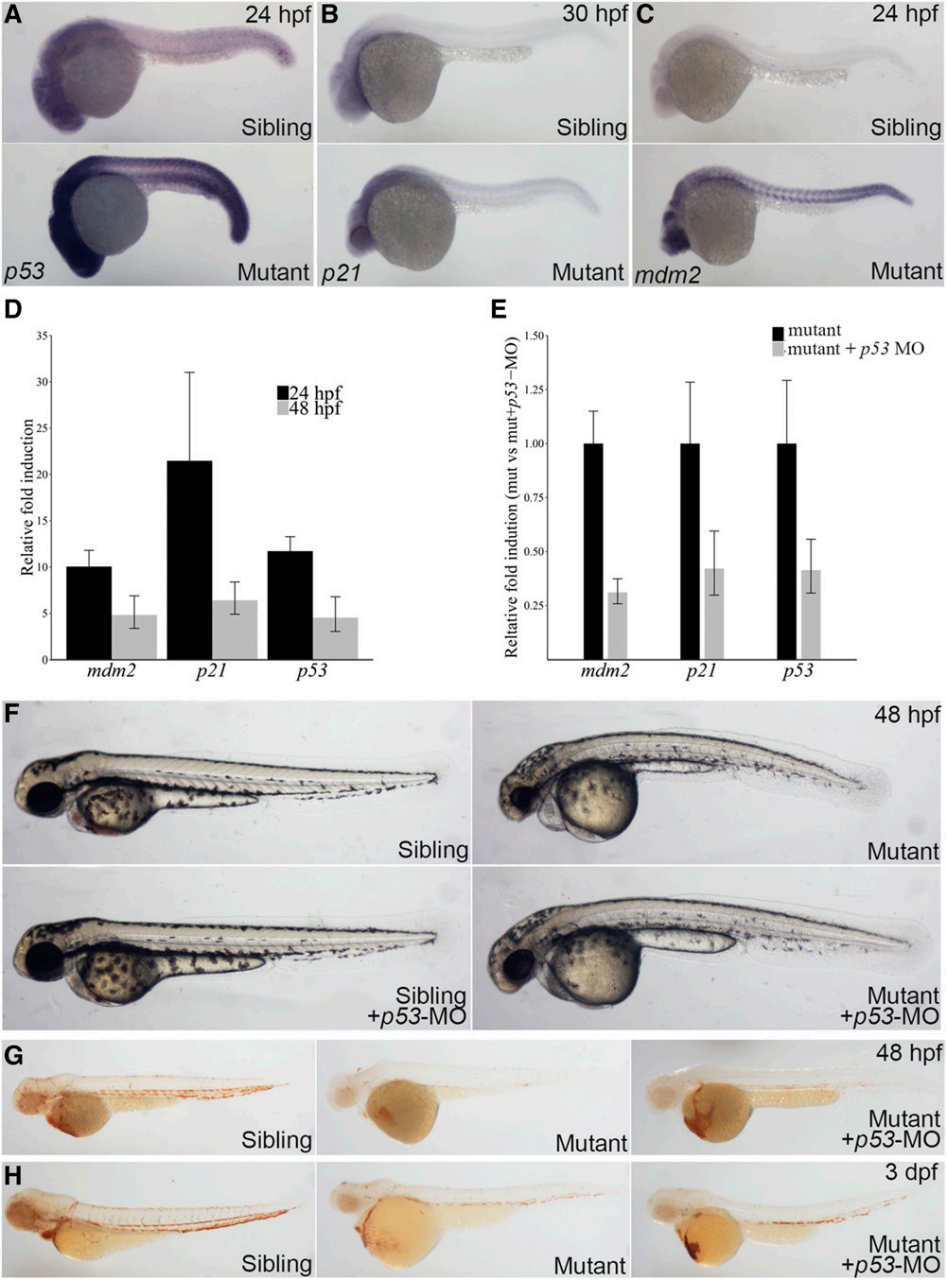
Anemic phenotype is partially rescued by *p53* konckdown in *rps9* deficient embryos. (A-C) Whole mount *in situ* shows that *p53*, *p21*, and *mdm2* are upregulated in mutants. (D) The expression of *p53* and *p53* target genes including *mdm2* and *p21* increases as measured by qPCR. The relative fold induction is represented as fold change over sibling control. (E) *p53* inhibition by MO injection knockdown the gene expression of *p53* and *p53*-target genes. (F) The morphological defects of *rps9* mutant are largely rescued by *p53* inhibition. (G-H) An increase of hemoglobin level appears at 48 hpf. (G) and 3 dpf (H) after *p53* knockdown.

The connection between elevated apoptosis and *p53* activation has been demonstrated in several ribosome protein mutants, and knockdown of *p53* can alleviate the phenotype (Danilova *et al.* 2008; Ear *et al.* 2016). Consistent with increased apoptosis level, expression of *p53* and *p53*-target genes was also increase, and this upregulation was observed throughout the entire embryo (Figure 5, A-D). By injecting morpholino (MO) targeting *p53* in *rps9* mutant, we analyzed whether down-regulating *p53* could ameliorate both the morphological and erythroid defects. Notable morphological improvement was found in the mutant followed by the decrease of *p53* and *p53*-target gene expression mediated by MO injection (Figure 5, E and F). Furthermore, erythropoiesis was also improved at 48 hpf and 3 dpf when detecting the hemoglobin level by o-dianisidine staining (Figure 5, G and H). Of note, no considerable changes to the blood level after *p53* inhibition in wildtype sibling control embryos (Figure S7). This suggests that the anemia phenotype is largely dependent on the *p53* pathway during both 48 hpf and 3 dpf in zebrafish *rps9* mutants.

### Rescue of anemia caused by rps9 deficiency using therapeutic agents

**Figure 6:**
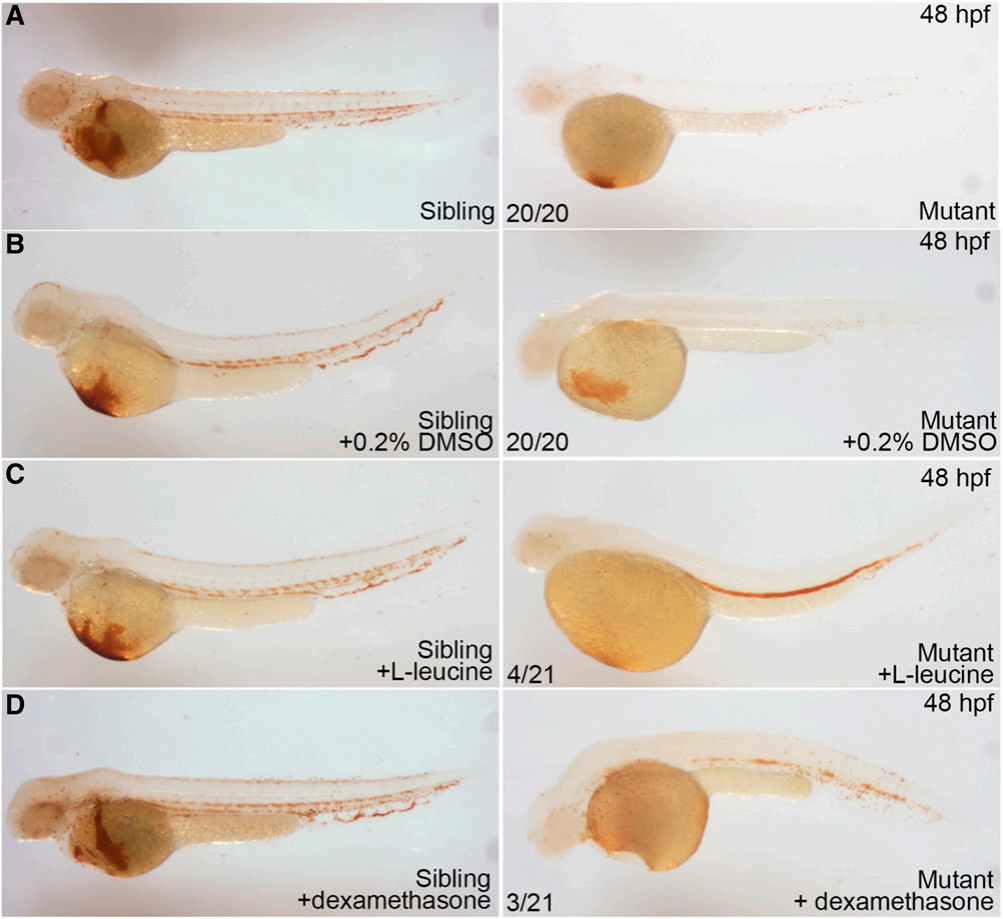
Hemoglobin level is partially recovered by L-leucine, and dexamethasone treatment in *rps9* deficient embryos. (A-B) Mutants without treatment and mutants treated with 0.2% DMSO were used as controls. (C) Mutants treated with L-leucine (200 mmol/L) show a considerable recovery in hemoglobin level. (D) Mutants treated with dexamethasone (250 μmol/L) display a less effective restoration in hemoglobin level.

L-leucine, and dexamethasone have been previously shown to alleviate anemia in ribosomal deficiency models (Danilova *et al.* 2011; Payne *et al.* 2012; Narla *et al.* 2014; Ear *et al.* 2016). As shown in Figure 6, treatment of embryos with L-leucine or dexamethasone notably recovered hemoglobin level in the mutant embryos (Figure 6). This result suggests that these agents might be used as an option of treatment for anemia if *rps9* mutation is identified in patients.

### Comparison of developmental defect of erythroid cells in four different ribosomal protein mutants

**Figure 7:**
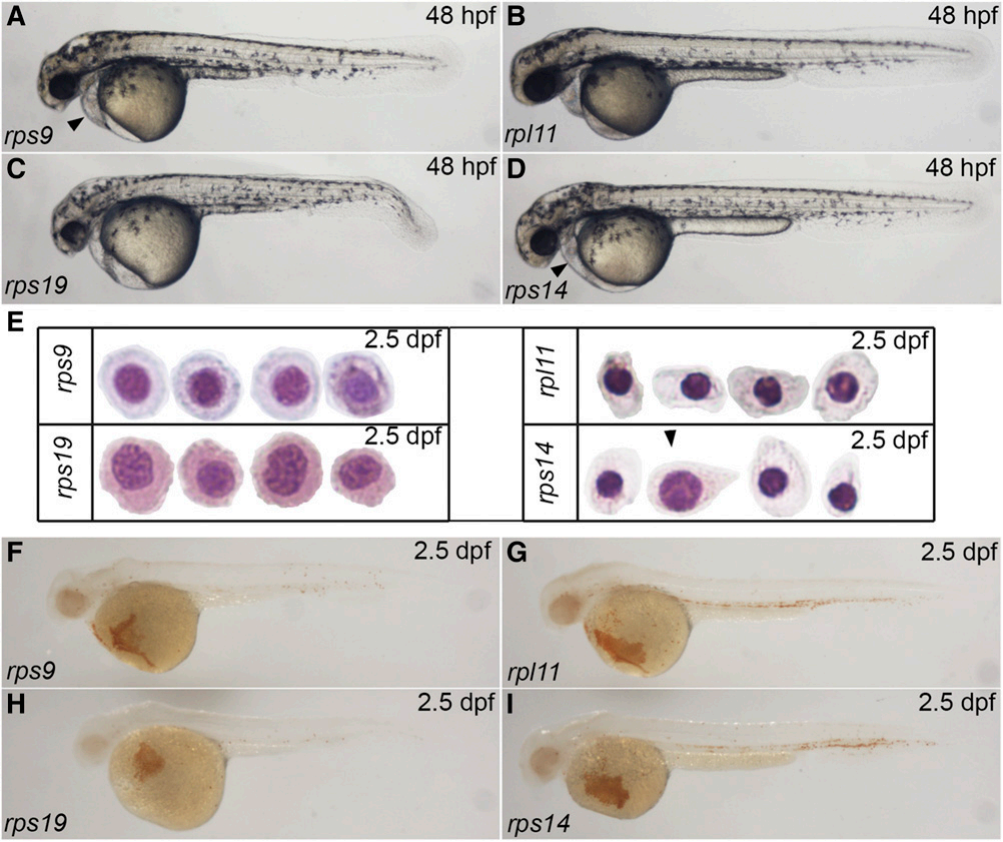
The severity of phenotype of four ribosomal protein mutants increases in an order of *rpl11*, *rps14*, *rps9*, and *rps19*. (A-D) Morphological comparison of four mutants by 48 hpf. Arrowhead indicates pericadial anema. (E) Wright-Giemsa staining for four mutants at 2.5 dpf. Arrowhead indicates that an erythrocyte in *rps14* mutants resembles the erythrocytes in *rps9* mutants. (F-I) o-dianisidine staining of four mutants at 2.5 dpf.

The developmental functions of ribosomal protein such as *rps19*, *rpl11*, and *rps14* have been previously reported in zebrafish (Danilova *et al.* 2011; Zhang *et al.* 2014; Ear *et al.* 2016). We compared the phenotype of these mutants with *rps9* mutant at the same developmental stage. At 48 hpf, *rps19* mutant displayed the most severe morphological defects, while *rpl11* mutant exhibited the mildest phenotype. The overall morphology of *rps9* mutant and *rps14* mutant appeared similar but *rps9* had a slightly shorter trunk and a larger pericardium (Figure 7, A-D). By 2.5 dpf, *rps9* mutant showed a less developed form of polychromatophilic erythroblast, whereas *rpl11* mutant displayed a more mature form, with *rps14* mutant being between them. At the same stage, erythroid cells in *rps19* mutant arrested at the basophilic stage, suggesting the maturation of erythrocyte is most severely impaired in *rps19* mutant (Figure 7E). Consistent with the cytomorphological change, the hemoglobin levels declined gradually in the order of *rpl11*, *rps14*, *rps9* and *rps19* mutants, implying that the status of erythrocyte maturation is correlated to anemic phenotype (Figure 7, F-I). In summary, this comparison illustrates that a variety of morphological abnormalities and anemic state are developed in different ribosomal protein mutants, thus requiring a detailed examination for each specific ribosomal protein mutants.

## Discussion

Through studying genetic mutation in zebrafish, we demonstrated that *rps9* plays an important role in erythropoiesis in zebrafish. Although no *rps9* mutation has been reported in any cases of anemia, this study suggests that *rps9* can be a potential candidate for DBA or other human anemia diseases. A recent genome-wide association study has implicated *rps9* as a candidate for Takayasu disease, which is a rare inflammatory disease that typically damages the aorta and its major branches (Renauer *et al.* 2015). To study whether *rps9* is involved in maintaining vascular structure, we analyzed the blood vessel with *flk* and *fli1*, two common vascular markers (Figure S8, A and B). A moderate disruption of intersomitic vessel formation was observed at 24 hpf in the mutant, implicating that *rps9* might be involved in the vessel formation in zebrafish. Similar to this observation, it was reported that *rps29* mutation also caused the disruption of vessel formation (Burns *et al.* 2009). However, *rpl11* mutation seemed not to cause any significant change of vessel morphology (Danilova *et al.* 2011). More experiments are needed to address the link between *rps9* and Takayasu disease or ribosomal deficiency and vascular integrity in general.

Anemia in *rps9* mutant is stronger than *rps14* mutant. Erythroid failure of *rps9* mutant appeared to be *p53*-dependent as early as 2 dpf. However, in *rps14* mutant, erythroid failure was demonstrated to be *p53*-dependent at 3 dpf (Ear *et al.* 2016). This timing difference seems to be correlated with the severity of erythroid failure.

Comparison of four zebrafish ribosomal protein mutants revealed that severity of morphological and anemic phenotype ranks in the order of *rpl11*, *rps14*, *rps9* and *rps19*. This raises the question why *rpl11* deficiency develops defects later than the deficiency in the other three ribosomal proteins. A recent study suggests that ribosomal stress is mediated by free ribosomal proteins, in particular by free RPL11, to bind MDM2 and subsequently activate P53 (Zheng *et al.* 2015). As mentioned before, *p53* activation induced by ribosomal protein disruption in zebrafish models has a great impact on the development of anemia, so it is possible that the other three mutants have more free RPL11 than *rpl11* mutant, thus accelerating P53 activation and showing more severe phenotype. Further experiments are needed to test this hypothesis.
